# miR-1303 regulates BBB permeability and promotes CNS lesions following CA16 infections by directly targeting MMP9

**DOI:** 10.1038/s41426-018-0157-3

**Published:** 2018-09-19

**Authors:** Jie Song, Yajie Hu, Hongzhe Li, Xing Huang, Huiwen Zheng, Yunguang Hu, Jingjing Wang, Xi Jiang, Jiaqi Li, Zening Yang, Haitao Fan, Lei Guo, Haijing Shi, Zhanlong He, Fengmei Yang, Xi Wang, Shaozhong Dong, Qihan Li, Longding Liu

**Affiliations:** 1 Institute of Medical Biology, Chinese Academy of Medical Science and Peking Union Medical College, Yunnan Key Laboratory of Vaccine Research and Development on Severe Infectious Diseases, Kunming, China; 2 Key Laboratory of Systemic Innovative Research on Virus Vaccine, Chinese Academy of Medical Sciences, Kunming, China

## Abstract

Coxsackievirus A16 (CA16) is a member of the Picornaviridae family and causes mild and self-limiting hand, foot, and mouth disease (HFMD) in infants and young children. CA16 infection can also progress to central nervous system (CNS) complications; however, the underlying mechanism by which CA16 penetrates the blood-brain barrier (BBB) and then causes CNS damage remains unclear. This study aimed to explore the mechanism of CA16 neurotropic tropism by establishing an in vitro BBB model with CA16 infection and an in vivo CA16 rhesus monkey infant infection model. The results showed that CA16 infection induced increased permeability of the BBB accompanied by upregulation of matrix metalloproteinase 9 (MMP9) expression. Subsequently, high-throughput miRNA sequencing technology and bioinformatics analysis revealed that miR-1303 may regulate BBB permeability by targeting MMP9. Next, we used dual-luciferase, qRT-PCR, and western blot assays to provide evidence of MMP9 targeting by miR-1303. Further experiments revealed that CA16 infection promoted the degradation of junctional complexes (Claudin4, Claudin5, VE-Cadherin, and ZO-1), likely by downregulating miR-1303 and upregulating MMP9. Finally, EGFP-CA16 infection could enter the CNS by facilitating the degradation of junctional complexes, eventually causing neuroinflammation and injury to the CNS, which was confirmed using the in vivo rhesus monkey model. Our results indicate that CA16 might penetrate the BBB and then enter the CNS by downregulating miR-1303, which disrupts junctional complexes by directly regulating MMP9 and ultimately causing pathological CNS changes. These results provide new therapeutic targets in HFMD patients following CA16 infection.

## Introduction

Coxsackievirus A16 (CA16), which belongs to the human enterovirus A species of the Picornaviridae family, is one of the major causative agents of hand, foot, and mouth disease (HFMD), which is a common infectious disease mostly seen in children under 5 years old^1^. In most instances, HFMD is a benign, self-limiting disease that is often characterized by fever, pharyngitis, and vesicular eruptions on the hands and feet, as well as in the mouth^2^. However, in some epidemic outbreaks, severe progressive HFMD with fatalities has been observed^3^. The occurrence of high mortality is usually concomitant with central nervous system (CNS) complications, such as acute flaccid paralysis, aseptic meningitis, brainstem encephalitis, and pulmonary edema^4^. Currently, three companies in mainland China, namely, Beijing Vigoo Biological Co., Ltd. (Vigoo), Sinovac Biotech Co., Ltd. (Sinovac), and the Chinese Academy of Medical Sciences (CAMS) have successfully developed inactivated EV71 vaccines. All three vaccines have been reported to have high safety and efficacy for preventing EV71 infections, but they have no cross-protective effects on other enterovirus infections, including CA16^5^. Previous studies have indicated that most CA16 infections present only mild symptoms. However, accumulating evidence in recent years has demonstrated that CA16 might actually be more virulent in children and has induced severe cases of CNS complications and fatal outcomes, which were similar to those of EV71 infections^6^. Furthermore, some severe and fatal HFMD cases with CA6 and CA10 infections have shown a clear increasing trend in recent years^7^. Taken together, these findings demonstrate that it is important to explore the underlying molecular basis of HFMD pathogenesis and progression induced by CA16 infections, which might provide novel approaches for the development of vaccines that cover multiple species of enterovirus.

Similar to poliovirus, CA16 is a highly neurotropic virus^8^. Two likely routes by which CA16 enter the CNS have been considered: the virus either is transmitted to the CNS from the blood across the blood–brain barrier (BBB) or enters the CNS through peripheral nerves via retrograde axonal transport^9,10^. Several studies have revealed that the BBB, which is primarily composed of brain endothelial cells, astrocyte end-feet, pericytes, perivascular macrophages, and a basal membrane, acts in concert with the immune system to restrict the entry of pathogens into the CNS. Meanwhile, many pathogens manage to cross the BBB via modifying a host of BBB functions, altering BBB integrity, and disrupting BBB permeability, which ultimately allows the establishment of infection within the CNS^11^. For example, West Nile virus enters the brain by enhancing the BBB permeability with matrix metalloproteinase 9 (MMP9) release. MMP9 is a member of the zinc-dependent and calcium-dependent endopeptidase family that is capable of degrading most extracellular matrix components^12^. Moreover, in the CNS, MMP9 can degrade components of the basal lamina and junctional complexes, leading to the disruption of BBB integrity and induction of the neuroinflammatory response in many neurological diseases^13^. For instance, alterations in BBB permeability were consistent with the increased expression of MMP9 in stroke and traumatic brain injury; further, it was found that disruption of BBB integrity in the setting of elevated MMP9 expression was closely associated with changes in junction assembly and function^14^. Junctional complexes in the BBB primarily include tight junctions (TJs), adhesion junctions (AJs), and gap junctions, which contribute differently to BBB dysfunction by directly regulating BBB permeability, altering transcellular exchange, affecting the expression pattern of certain transporters, and modifying endothelial metabolic processes^15^. Hence, destruction of these junctional complexes indirectly mirrors the breakdown of BBB integrity.

Our previous study analyzed transcriptome changes in a CA16-infected rhesus monkey model and found that the expression of MMP9 was significantly increased^16^. Thus, we speculated that the CNS injuries induced by CA16 infection are derived from direct penetration of the BBB by CA16 and that the molecular mechanism of BBB penetration by CA16 is related to the deregulation of junctional complexes via MMP9. Furthermore, we aimed to explore the upstream regulatory factors of MMP9 using miRNA high-throughput sequencing technology.

## Results

### Proliferation kinetics of CA16 in HUVECs

To assess whether CA16 can infect and proliferate in HUVECs, the cell line was inoculated with each virus and analyzed for proliferation kinetics; EV71 experiments were performed in the same manner as the control. The level of EV71 and CA16 viral load showed a constant rise over time (Fig S1A). Moreover, the production of infectious virus particles gradually increased over time with EV71 and CA16 infections (Fig S1B), suggesting that HUVECs are highly susceptible to EV71 and CA16.

### CA16 infections promote HUVEC permeability

**Fig. 1:**
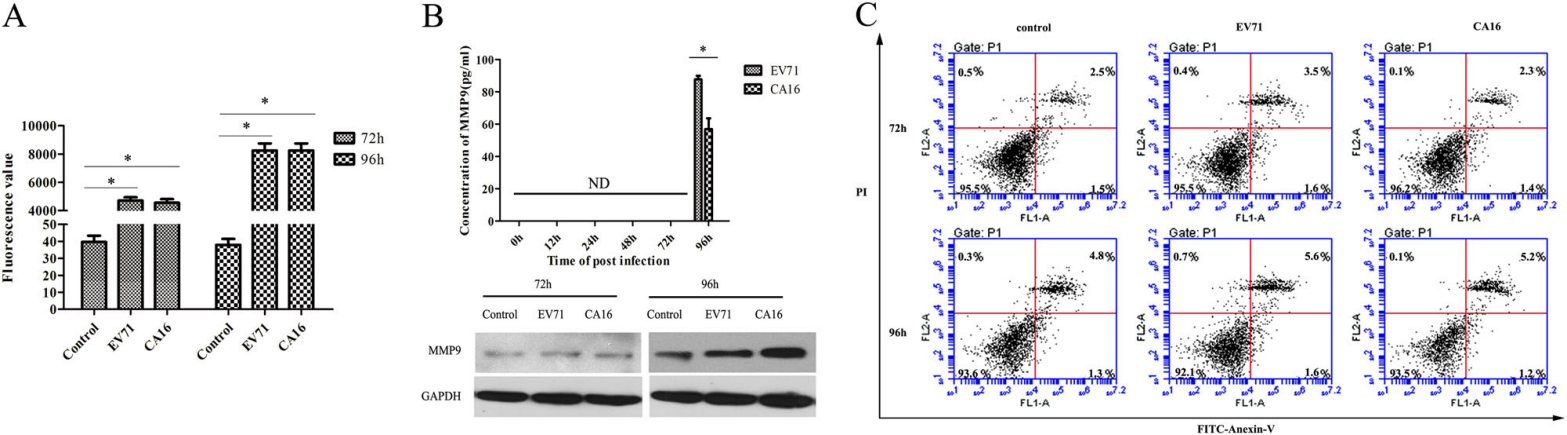
CA16 infections increased permeability of monolayer HUVECs. **a** Permeability of monolayer HUVECs was detected by a paracellular FITC-dextran flux assay. Significant differences among these groups are indicated by *P < 0.05. **b** Detection of the expression of MMP9 induced by CA16 infection using ELISA and WB. **c** CA16 infections does not induced HUVECs apoptosis, including early apoptotic and late apoptotic

To determine the effects of CA16 infection on the integrity of HUVECs, we performed a HUVEC endocytosis assay using FITC-dextran. The results revealed that FITC-dextran leakage was significantly upregulated in CA16-treated HUVECs (Fig. 1a), indicating that CA16 infections might enhance cell permeability. Coincidently, CA16 infection induced increased permeability of the BBB that was accompanied by upregulated MMP9 expression (Fig. 1b). Subsequently, an apoptosis assay was used to evaluate whether the alterations in HUVEC permeability were attributed to apoptosis (Fig. 1c). There were no significant differences in HUVEC apoptosis induced by CA16 infections, implying that apoptosis might not be responsible for HUVEC permeability during CA16 infection. Similar results were observed in EV71-infected HUVECs.

### Global analysis of miRNA sequencing data in HUVECs following CA16 and EV71 infections

**Fig. 2:**
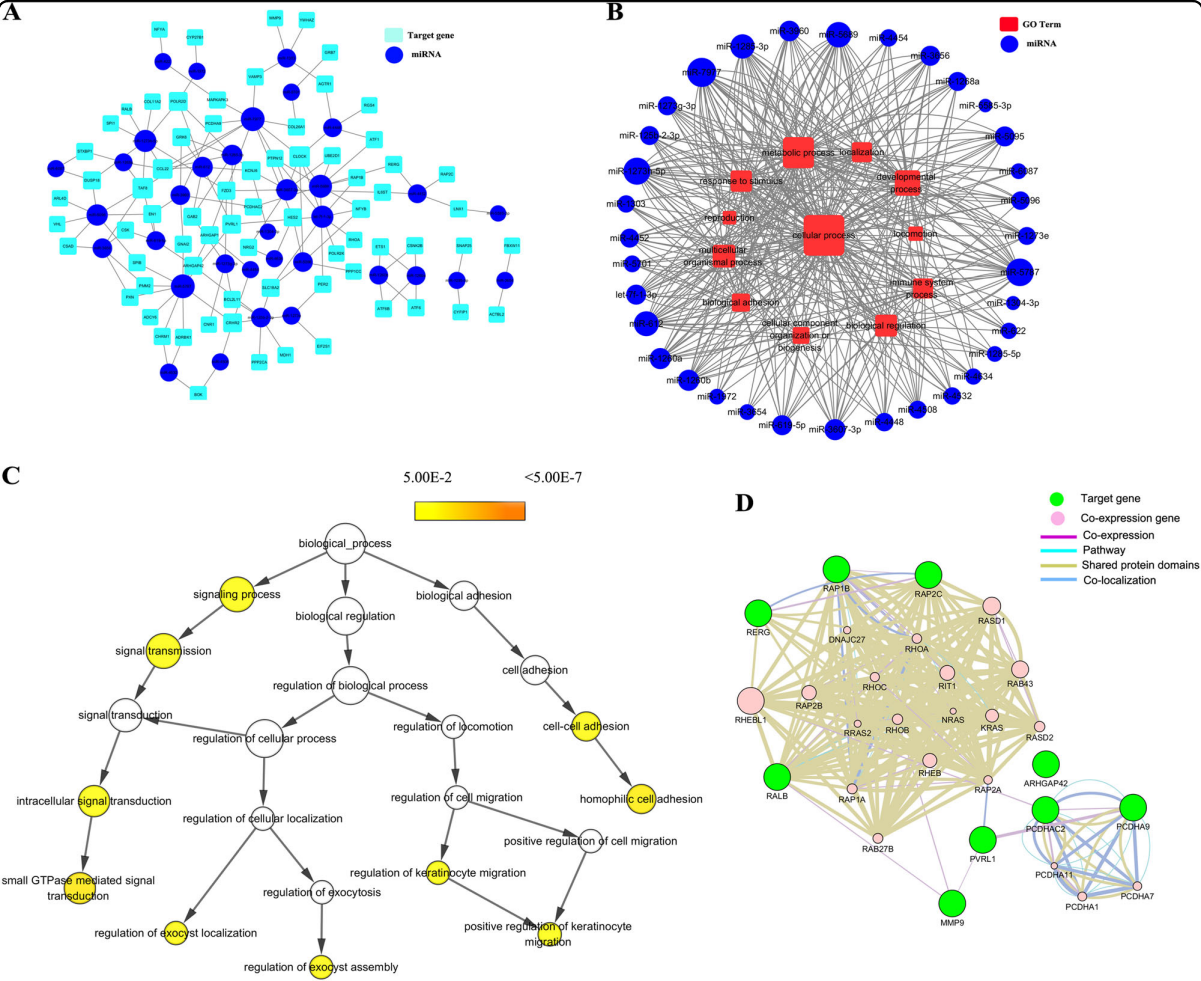
Predicted networks between the coincident trend expressed miRNAs and their putative target genes, their putative target gene-associated GOs, Hierarchical tree graphs of GO terms and co-expression network. All miRNAs are depicted as a blue colored node among the networks. The larger the area of the nodes, the bigger the number of connections between a miRNA and other nodes in the network.**a** Predicted network between the same expressed miRNAs and their putative target genes. Putative targets are presented wathet blue rounded rectangles. The width of the line represents the free energy between the miRNAs and their putative target genes. **b** Predicted network between the same expressed miRNAs and their putative target gene-associated GOs. Red rectangle nodes denote GOs. Edges show the inhibitory effects of miRNAs on GOs. **c** In total, 71 genes were used to generate the GO tree. Edges represent “parent–child” relationships of GO terms. The sizes of the yellow rounded rectangles are proportional to the number of GO terms annotated to each node. **d** Co-expression network were constructed by the top ten significantly putative targets. Green nodes are target genes and pink nodes are co-expression genes. Genes with bigger size are more centralized in the network and have a stronger capacity of modulating adjacent genes. Different color lines mean the different interactions between these genes

Because CA16 and EV71 both caused remarkable changes in HUVEC permeability, we hypothesized that CA16 and EV71 infections might induce similar alterations. Thus, in this study, we screened the differentially expressed miRNAs, which showed the same expression pattern in the trend analysis (Fig S2). Subsequently, the 34 miRNAs expressed at the same trend were used to predict potential targets via TargetScan and the miRDB algorithm. As shown in Fig S3, the 658 overlapping targets identified by both programs were considered key targets of these miRNAs for the following GO and pathway analysis. Moreover, the GO results revealed that these target genes were categorized as follows: twelve as a biological process (Fig S4A), eight as a molecular function (Fig S4B), and eight as a cellular component (Fig S4C). Then, the pathway results revealed 79 pathways (Fig S5). To further narrow down the key targets, we identified intersections of related genes in the GO and pathway analyses, and 71 targets were identified, as shown in Fig S6. Consequently, a miRNA–gene network (Fig. 2a) and miRNA–GO network (Fig. 2b) were built based on interactions between miRNAs and genes or miRNAs and GOs. Furthermore, these genes were used for a hierarchical GO category and coexpression network analysis. The results uncovered that signaling process, signal transmission, intracellular signal transduction, small GTPase-mediated signal transduction, regulation of exocyst localization, regulation of exocyst assembly, cell–cell adhesion, regulation of keratinocyte migration, and positive regulation of keratinocyte migration were significantly different (Fig. 2c). Moreover, coexpression results revealed that junctional complexes, such as PCDHA11, PCDH1, and PCDHA7, were coexpressed. Therefore, these findings suggested that the deregulated miRNA-associated target genes might be involved in cell junctions. In addition, junctional complexes of the coexpression network contained MMP9 molecules (Fig. 2d), which is an important factor that allows some neurotropic viruses to cross the BBB. However, our results also revealed that CA16 and EV71 infections might enhance BBB permeability. Therefore, we focused our attention on MMP9. To seek the major miRNA regulator of MMP9, we focused on the miRNA-target network and found that the only miRNA that interacted with MMP9 was miR-1303. Hence, we speculated that the abnormal expression of miR-1303 induced by CA16 and EV71 infections might enhance the increased BBB permeability via regulating MMP9 expression.

### miR-1303 directly targets MMP9 and degrades junctional proteins

**Fig. 3:**
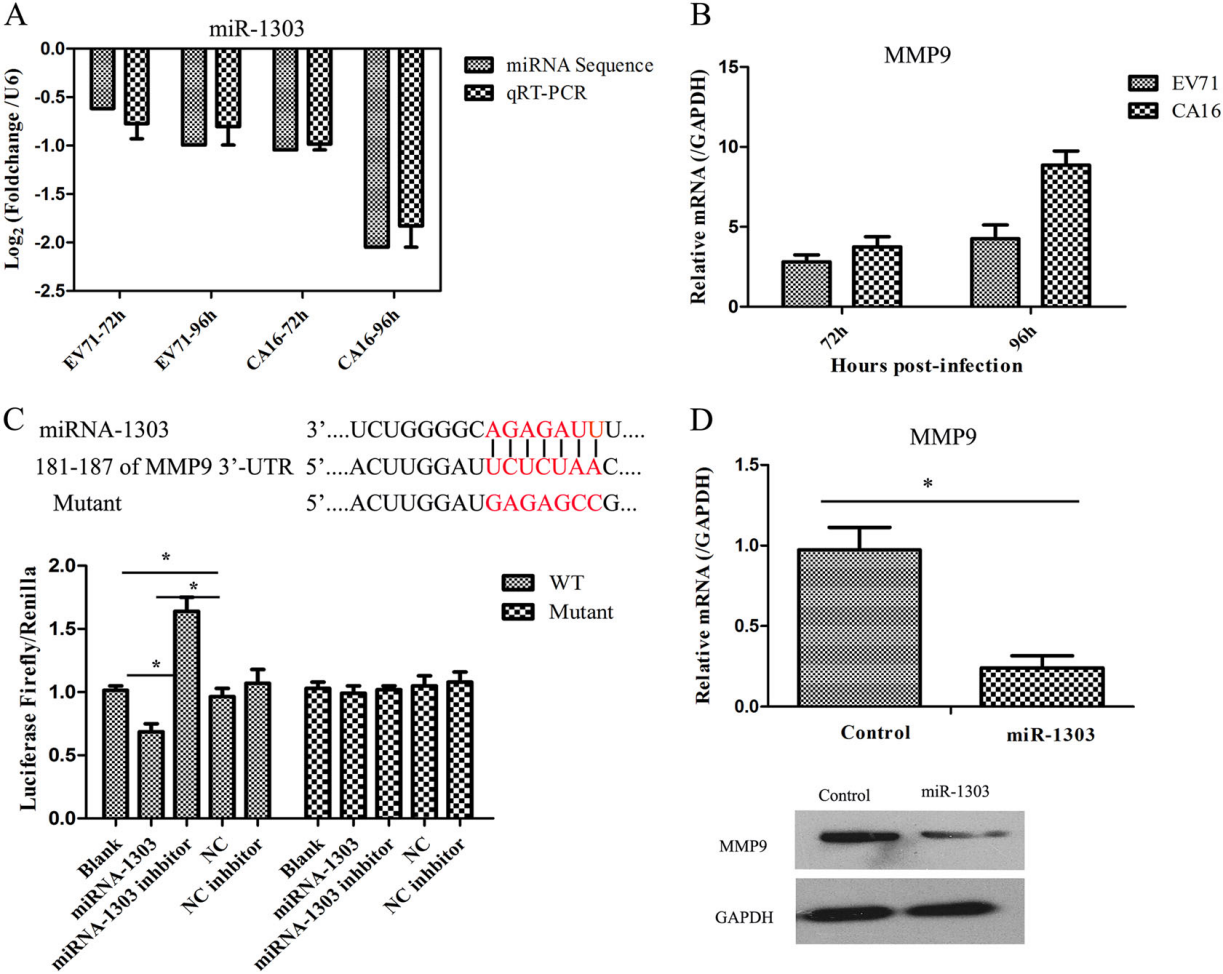
MMP9 is a target gene of miR-1303. **a** Verification of the expression of miR-1303 by qRT-PCR compared to miRNA sequencing. **b** Detection of the expression of MMP9 induced by CA16 infection using qRT-PCR. **c** Dual-luciferase reporter analysis verified the targetting relationship between miR-1303 and MMP9. **d** qRT-PCR and WB analysis of MMP9 expression in miR-1303 transfected cells. Error bars represent the mean ± SEM, and the data are averages from three biological replicates, *P < 0.05

qRT-PCR was applied to detect miR-1303 and MMP9 expression levels in HUVECs following EV71 and CA16 infections. The level of miR-1303 was markedly decreased, which was consistent with our miRNA sequence results (Fig. 3a), whereas the level of MMP9 was remarkably increased (Fig. 3b). Subsequently, based on the inverse correlation between miR-1303 and MMP9 expression, we further explored the target interaction between miR-1303 and MMP9 by a dual-luciferase reporter assay. The results revealed that the luciferase activity was notably decreased in the WT-MMP9 vector transfected with miR-1303 mimics compared with that in the other four groups, while the luciferase activity was not markedly changed in the mutant-MMP9 vector transfected with the five plasmids (Fig. 3c). We further assessed the influence of miR-1303 on the expression of MMP9 by detecting the mRNA and protein levels after transfecting miR-1303 mimics and found that forced miR-1303 expression resulted in a sharp decline of MMP9 mRNA and protein levels in 293T cells (Fig. 3d). Hence, these data demonstrated that miR-1303 might directly target MMP9.

**Fig. 4:**
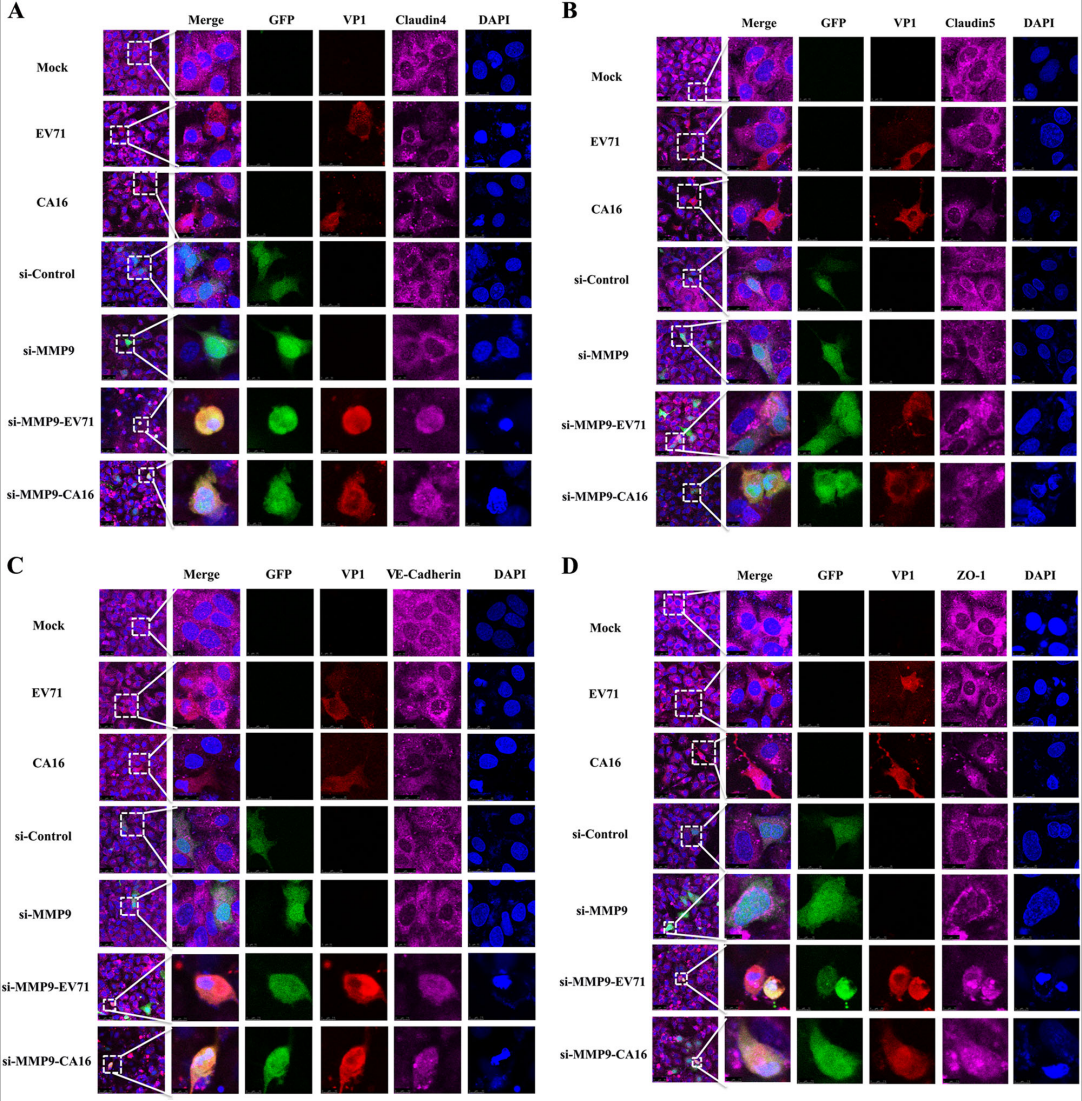
Confocal imaging showed that the junctional protein including **a** Claudin4, **b** Claudin5, **c** VE-Cadherin, and **d** ZO-1 were degraded by CA16 infection and knockout MMP9 rescued these degradation. The si-control and si-MMP9 plasmids contain a GFP tag (green color). EV71/CA16-VP1, junctional proteins and the cell nucleus are indicated by red, purple and mazarine, respectively

**Fig. 5:**
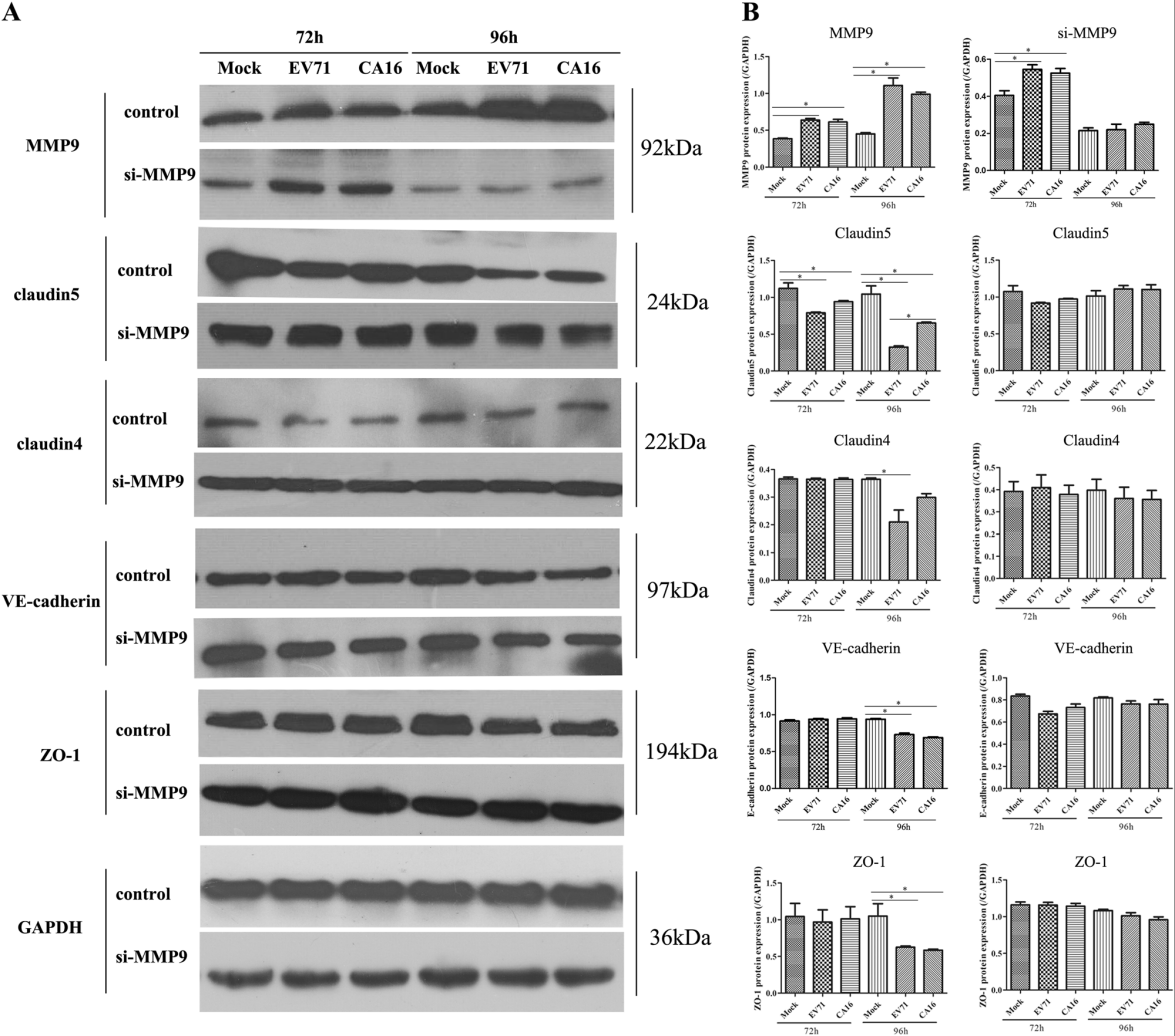
Junctional proteins expression including Claudin4, Claudin5, VE-Cadherin, and ZO-1 were degraded by CA16 infection and knockout MMP9 rescued this degradation. **a** Protein expression of junctional proteins in HUVECs with different treatments normalized against GAPDH. **b** Densitometry of WBs of three independent experiments performed with lysates at 72 and 96 hpi. The signal intensity was normalized to GAPDH levels from the same blots. The results are expressed as the mean ± SEM for three subjects, *P *<* 0.05

**Fig. 6:**
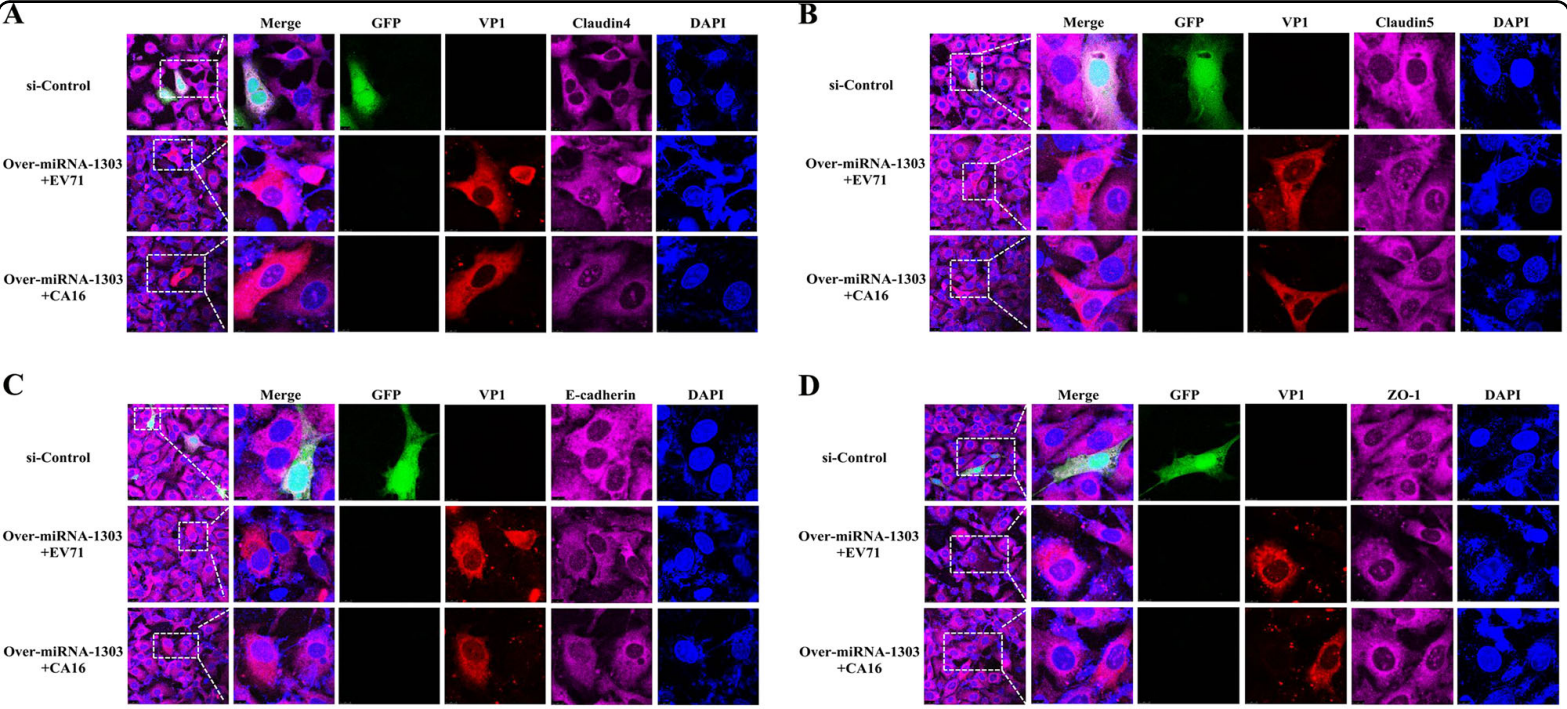
Overexpression of miR-1303 inhibites the degradation of junctional protein including **a** Claudin4, **b** Claudin5, **c** VE-Cadherin, and **d** ZO-1. EV71/CA16-VP1, junctional proteins and the cell nucleus are indicated by red, purple and mazarine, respectively

Since junctional proteins play a major role in the regulation of BBB permeability, and their disruption is a hallmark of CNS abnormalities, we determined whether miR-1303 might regulate the expression of junctional proteins in HUVECs through MMP9. Based on the above results, which showed that MMP9 expression was upregulated in HUVECs following CA16 and EV71 infections, we prepared plasmids for MMP9 depletion (si-MMP9) and found that the protein knockdown efficiency of si-MMP9 was approximately 50% compared to the Mock and si-control in HUVECs (Fig S7). As illustrated in Fig. 4a, cells in the mock, si-control, and si-MMP9 groups showed the normal “chicken wire” appearance of the Claudin4 structure. Compared with that in CA16-infected and EV71-infected cells, which displayed an obscure localization of Claudin4 at the cell–cell contact sites, Claudin4 staining was markedly enhanced in the si-MMP9-CA16 and si-MMP9-EV71 groups (Fig. 4a). Subsequently, IF staining was performed for other junctional proteins, such as Claudin5, VE-Cadherin, and ZO-1. The location of these proteins presented an alteration similar to that of Claudin4 in each group (Fig. 4b–d). Therefore, these observations indicated that CA16 and EV71 infections could interfere with junctional protein assembly in HUVECs, which might be intimately linked to breakdown of the BBB because of the upregulation of MMP9. However, knockdown of MMP9 could maintain the locations of junctional proteins in CA16-infected and EV71-infected HUVECs. Furthermore, western blot results also confirmed that CA16 and EV71 infections in HUVECs could upregulate MMP9 protein levels and downregulate the previously mentioned junctional protein levels, whereas these results were reversed when CA16-infected and EV71-infected HUVECs were transfected with a si-MMP9 plasmid (Fig. 5). Finally, according to the target relationship of miR-1303 and MMP9, we further detected the influences of miR-1303 at the locations of junctional proteins in CA16-infected and EV71-infected HUVECs. After transfection with a miR-1303 overexpression plasmid, Claudin4, Claudin5, VE-Cadherin, and ZO-1 proteins were not destroyed in EV71-infected and CA16-infected HUVECs (Fig. 6). Collectively, these results implied that miR-1303 might target MMP9 to inhibit junctional protein degradation, which might protect the BBB from damage.

### MMP9 promotes the entry of CA16 into the CNS and exacerbates CNS lesions by degrading junctional proteins in vivo

**Fig. 7:**
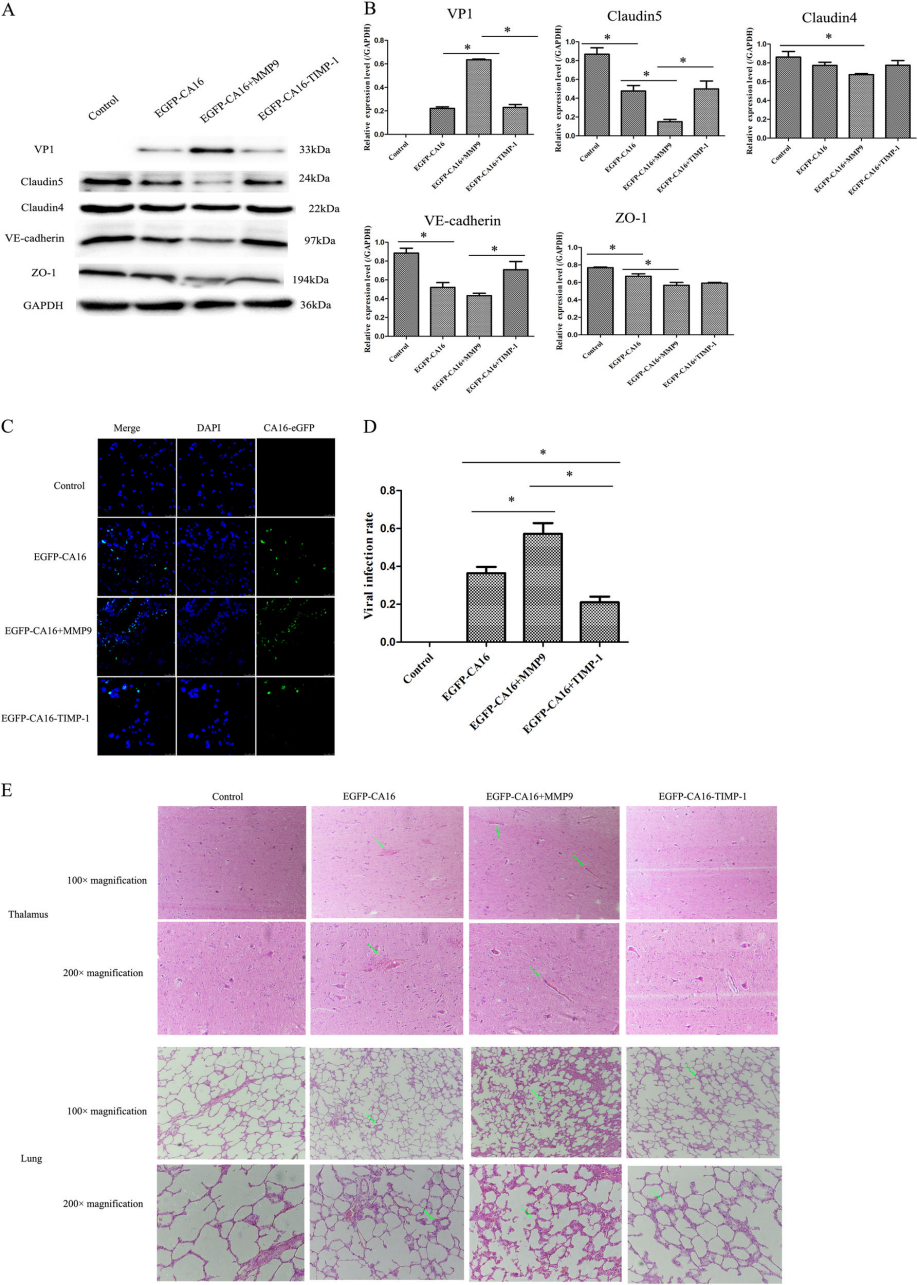
MMP9 promotes the degradation of junctional protein and CA16 entry into the CNS, which leading to CNS lesion in CA16-infected rhesus monkeys. **a** Junctional protein expression in thalamus of different treated rhesus monkeys. **b** Statistical analysis of the quantitative value of intensity about the blots. The ratios indicated the intensity of the bands compare to the GAPDH. **c** The efficacy of CA16 infections in thalamus of different treated rhesus monkeys. CA16-VP1 and the cell nucleus are indicated by green and mazarine, respectively. **d** Statistical analysis of viral infection rates. The ratios indicated the infected cells compared to the total cells from ten different visual fields. **e** Histopathological changes in the thalamus and lungs of different treated rhesus monkeys. The green arrow points to a place where pathological changes are obvious

In a CA16-infected rhesus monkey model, levels of junctional proteins, including Claudin5, VE-Cadherin, and ZO-1, were significantly reduced in EGFP-CA16-infected monkeys compared with control monkeys. Although Claudin4 did not present a notable reduction, it exhibited a decrease trend in EGFP-CA16-infected monkeys compared to control monkeys. In addition, these protein levels were further reduced in EGFP-CA16 + MMP9-infected monkeys compared with EGFP-CA16-infected monkeys. However, the expression of these proteins was notably compensated in EGFP-CA16 + TIMP-1-infected monkeys relative to EGFP-CA16 + MMP9-infected monkeys (Fig. 7a, b). Therefore, these data suggested that MMP9 plays a key role in degrading junctional proteins in HUVECs. The viral infection rates of brain tissues and viral genomic copies in EGFP-CA16 + MMP9 monkeys were the highest, followed by those in EGFP-CA16 monkeys, and they were the lowest in EGFP-CA16 + TIMP-1 monkeys (Fig. 7c, d and S8). This result indicated that MMP9 accelerates CA16 entry into the brain. Ultimately, the morphological examination showed no obvious pathological changes in the thalamus or lungs of control monkeys, whereas different pathological responses to EGFP-CA16 infection and EGFP-CA16 + MMP9 and EGFP-CA16 + TIMP-1 treatments were found in the thalamus and lungs. As presented in Fig. 7e, mild hemorrhagic injuries were observed in the thalamus tissues of the EGFP-CA16 monkeys; serious hemorrhagic injuries were discovered in the thalamus tissues of the EGFP-CA16 + MMP9 monkeys, but these morphological changes were drastically improved in the EGFP-CA16 + TIMP-1 monkeys. Additionally, the alveolar wall in the EGFP-CA16 monkeys and EGFP-CA16 + MMP9 monkeys was visibly thicker than that in the control monkeys (Fig. 7e). Nevertheless, the alveolar wall in the EGFP-CA16 + TIMP-1 monkeys was not apparently thickened (Fig. 7e). Therefore, these findings indicate that MMP9 may exacerbate CNS lesions and lung damage.

## Discussion

Epidemics of CA16 infections have caused many fatalities from serious CNS complications in the Asia-Pacific region since 1997 and have become a serious public health problem in these areas^17^. Nevertheless, the mechanism of CNS damage induced by CA16 remains obscure. MMPs are broad, nonspecific extracellular matrix-degrading enzymes that comprise collagenases (MMP1, 8, and 13), gelatinases (MMP2 and 9), stromelysins (MMP3, 10, and 11), and matrilysins (MMP7 and MMP26). Previous studies have shown that the increased MMP2 and MMP9 concentration in cerebrospinal fluid degrades junctional complex proteins between brain capillary endothelial cells, which further causes the disruption of BBB functions and ultimately promotes the formation of CNS-related diseases, such as ischemic stroke, multiple sclerosis, and glioblastoma^18^. The BBB is nonfenestrated to large molecules or xenobiotics, thus providing protection against invasion of the CNS by macromolecules and microorganisms^19^. The integrity of the BBB is a result of a tightly sealed monolayer of brain microvascular endothelial cells (BMECs), with TJs and AJs forming the seal between cells at junctional complexes^20^. Furthermore, many viruses (e.g., mouse adenovirus type 1) can trigger changes in BBB permeability by direct disruption of the TJ complex in endothelial cells^21^. Additionally, evidence is emerging that MMP9 plays an important role in the pathogenesis of BBB breakdown through regulating the complex intercellular junctional structure in BMECs^22^. For example, the HIV-1-specific protein Tat enhances the production of MMP9, which can decrease TJ protein expression and finally result in an increase of BBB permeability^22^. Yang et al.^23^ recently speculated that miR-3473a is involved in destruction of the BBB by targeting MMP9 during EV71 infections based on miRNA expression profiles, but this speculation lacks further experimental support. In our study, it was first found that CA16 could increase the permeability of HUVECs in an in vitro BBB model. Previous research has shown that Japanese encephalitis virus is a neurotropic flavivirus that can compromise the structural and functional integrity of the BBB, likely via enhancing the viral load in BMECs, and has the potential to subvert host cell apoptosis to optimize the course of the viral infection through deactivation of pro-apoptotic proteins. This suggests that apoptosis induced by the virus and occurring in BMECs might promote breakdown of the BBB^24^. Nevertheless, the BBB opening in this study was not related to cell apoptosis induced by CA16 infections (Fig. 1). Furthermore, HUVECs are an important constituent of the BBB in an in vitro model^25^. Therefore, to further characterize the mechanism by which CA16 modulates the upregulation of BBB permeability, we performed a miRNA profile analysis with high-throughput sequencing technology in CA16-infected HUVECs.

miRNAs are known to play a role in the replication and propagation of viruses, including cellular antiviral responses and/or the promotion of viral infections through complex regulatory pathways^26^. In this study, we screened the differentially expressed miRNAs in HUVECs after CA16 infection, focusing our attention on the similarly expressed miRNAs (Fig S2). Meanwhile, the potential targets of these miRNAs were predicted by TargetScan and miRDB (Fig S3). Overall, 658 intersection targets were used to further analyze the GO and pathways. The results of this analysis revealed that there were many intercellular adhesion-related GOs and pathways, such as biological adhesion, cell junction, and cadherin signaling pathways (Fig S4). Subsequently, the overlaying targets involved in both GOs and pathways were applied to construct miRNA-regulatory networks and gene coexpression networks. The results showed that MMP9 was only targeted by miR-1303, which was also implicated in the regulation of biological adhesion (Fig. 2a–c). In addition, there was an interaction between MMP9 and PVLR1 in a coexpression network (Fig. 2d). PVRL1, also known as nectin1, was first identified as an afadin-binding protein and serves as a cell adhesion molecule at AJs^27^. However, the formation of TJs in BMECs generally requires the existence of AJs. Thus, according to the above clues, we hypothesized whether the increased BBB permeability during EV71 and CA16 infections was attributed to the downregulation of miR-1303, which might negatively regulate the secretion of MMP9 and eventually lead to alterations in the junctional complexes between HUVECs.

To address this hypothesis, it was necessary to confirm the expression levels of miR-1303 and MMP9 in an in vitro model of the BBB. miR-1303 and MMP9 expression was detected by qRT-PCR, ELISA, and western blot. It was observed that miR-1303 expression was reduced, whereas MMP9 expression was elevated in HUVECs following CA16 infections, which was consistent with the high-throughput sequencing data (Fig. 3a, b). Moreover, the dual-luciferase reporter assay directly indicated that MMP9 was the target gene of miR-1303 (Fig. 3c). Additionally, the downregulated expression of MMP9 at the mRNA and protein levels further demonstrated the target interaction between miR-1303 and MMP9 by overexpression of miR-1303 in 293T cells (Fig. 3d). To date, the investigations between miR-1303 and disease have mainly focused on cancers. For example, Zhang et al.^28^ verified that miR-1303 facilitates the proliferation, migration, and invasion of gastric cancer through targeting claudin-18. However, the role of miR-1303 in CNS-related diseases has not been reported; therefore, based on the target interaction between miR-1303 and MMP9, we aimed to further investigate the effects of miR-1303 on changes in BBB permeability in HUVECs following CA16 infections.

Junctional complexes between BMECs, such as TJs and AJs, play a crucial role in the regulation of BBB permeability^15^. TJs are the luminal-most cellular junction and are composed of claudins, occludin, and zona occludens, which seal the paracellular route between BMECs, creating a high transendothelial cell electrical resistance barrier that impedes ions and small charged molecules from crossing the BBB. However, AJs are positioned abluminal to TJs and consist of VE-cadherin dimers that mediate cell–cell membrane adhesion and bind to the actin cytoskeleton via catenins in BMECs^29^. For instance, rabies virus infection promotes the release of a large number of inflammatory cytokines, which further reduces the expression of TJ proteins in the vascular endothelial cells of the BBB and ultimately results in increased BBB permeability^30^. Thus, maintaining the intact structure and function of TJ-associated and AJ-associated proteins is essential for BBB integrity. In our study, the expression levels of TJ-associated proteins Claudin4, Claudin5, and ZO-1, as well as the AJ-associated protein VE-cadherin, were significantly reduced in HUVECs after infection with CA16 (Fig. 4). Moreover, the expression levels of these proteins were not remarkably affected in CA16-infected HUVECs that were pretransfected with a si-MMP9 plasmid (Fig. 5). Claudin4, Claudin5, and ZO-1 are often considered sensitive indicators of normal and disturbed functional states of the BBB, while VE-cadherin, a member of a transmembrane Ca^2+^-dependent adhesion molecule family, regulates vascular permeability in the BBB^31^. Therefore, our results suggested that CA16 infections result in BBB dysfunction likely through modulating the expression of TJ-associated and AJ-associated proteins, which might be intimately related to MMP9. MMP9 is normally only present at low levels in the brain tissue and cerebrospinal fluid but is markedly upregulated in many infectious diseases of the CNS^32^. Subsequently, based on the target interaction between miR-1303 and MMP9, we continued to investigate the effects of miR-1303 on the above junctional proteins^21^. The results revealed that the expression levels of Claudin4, Claudin5, ZO-1, and VE-cadherin were not notably reduced in CA16-infected HUVECs pretransfected with a miR-1303 plasmid (Fig. 6). Hence, this result indicated that the downregulation of miR-1303 induced by CA16 infections in HUVECs reduced the expression levels of Claudin4, Claudin5, ZO-1, and VE-cadherin by accelerating the release of MMP9, which might explain why the BBB permeability increased during EV71 and CA16 infections.

To further confirm whether the degradation of junctional complexes could facilitate entry of CA16 into the CNS by breaking the BBB, which eventually leads to CNS damage, we conducted in vivo animal experiments (Fig. 7a). The viral infection rate in EGFP-CA16 + MMP9 monkeys was higher than that in EGFP-CA16 monkeys and further higher than that in EGFP-CA16 + TIMP-1 monkeys (Fig. 7c and Fig S8). Thus, these results confirmed that the brain tissues might have been susceptible to CA16 infection mainly because of the effects of MMP9 on BBB permeability, which eventually caused more severe CNS damage. Moreover, morphological examination revealed that the neuropathological lesions were the most marked in brain tissues of EGFP-CA16 + MMP9 monkeys, with obvious hemorrhagic injuries. Nevertheless, the above pathological changes were mild in the EGFP-CA16 monkeys and were not apparent in the EGFP-CA16 + TIMP-1 monkeys (Fig. 7e).

**Fig. 8:**
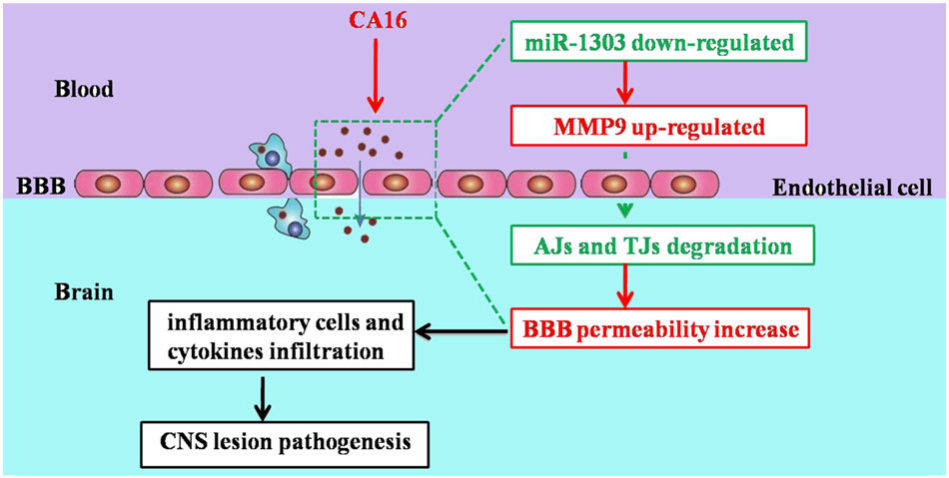
Summary of the CA16-infected CNS lesion process. CA16 penetrates the BBB and enter the CNS by downregulating miR-1303, which induced the disruption of junctional complexes by directly regulating MMP9 and ultimately caused the pathological CNS changes

Our results are the first to demonstrate that CA16 infections in HUVECs can decrease the expression of miR-1303, which relieves the inhibitory role in MMP9 release and thus promotes MMP9 expression. Therefore, increased MMP9 expression enhanced the BBB permeability by directly degrading junctional complexes, which allowed the virus to enter the CNS and ultimately led to CNS lesions (Fig. 8). We will further consider verifying this conclusion in humanized mice or genetically modified rhesus monkeys. These results might illustrate the potential molecular mechanism of BBB permeability during CA16 infections. This study not only provides a target for the clinical treatment of CA16 infections but also offers an early warning molecule for severe HFMD patients.

## Materials and methods

### Ethics statement and animal experiments

All animal experimentation was performed with the approval of the Yunnan Provincial Experimental Animal Management Association (Approval number: SCXK [Dian] 2011-0005), the Experimental Animal Ethics Committee of the institute (Approval number: YISHENGLUNZI [2011]15), and the Experimental Animal Administration and Ethics Committee of the Institute of Medical Biology, Chinese Academy of Medical Sciences (IMBCAMS) (Approval number: [2017]16) and in accordance with the principles of “Guide for the Care and Use of Laboratory Animals” and “The Guidance to Experimental Animal Welfare and Ethical Treatment.”

The rhesus monkeys were reared separately in a large cage (BSL-2 conditions) with sufficient fresh air and natural light, which allowed visual, olfactory, and auditory interactions with other monkeys. Each room had a temperature control valve guaranteed to maintain the room temperature at ~25 °C, and food, water, and fruit were readily available. Appropriate treats and vitamins were provided. The animals were given access to environmental enrichment (such as approved toys) to promote their psychological wellbeing. All animals were fully under the care of veterinarians at IMBCAMS.

A CA16-infected rhesus monkey model was generated by nasal spray using 5 lgCCID_50_/100 µL EGFP-CA16, while two rhesus monkeys were given MMP9 (150 ng/100 μL) or TIMP-1 (150 ng/100 μL) (an MMP9 inhibitor) recombinant proteins through intravenous injection every 2 days. One rhesus monkey injected with phosphate-buffered saline (PBS) served as a control. After 6 days, all rhesus monkeys were euthanized using ketamine as the anesthetic (10 mg/kg of body weight), and samples were collected for viral load examination, morphological analysis, and junctional protein evaluation.

### Cell culture and virus infection

Human umbilical vein endothelial cells (HUVECs) purchased from the American Type Culture Collection (Manassas, USA) were maintained in Dulbecco’s modified Eagle’s medium (Corning, USA) supplemented with 10% fetal bovine serum (Gibco, USA), 2 mmol/L L-glutamine, 100 U/mL penicillin, and 100 µg/mL streptomycin at 37 °C in a humidified atmosphere containing 5% CO_2_. Strains EV71 (subgenotype C4, GenBank: EU812515.1) or CA16-G20 (subgenotype B, GenBank: JN590244.1) were respectively isolated from an epidemic in Fuyang, China in 2008 and from an HFMD patient in Guangxi, China in 2010 and were used throughout the study. The HUVECs were cultivated in 6-well plates to 80% confluence and then infected with EV71 or CA16 at a multiplicity of infection (MOI) of 0.1. The infected cells were collected at 0, 72, and 96 h post infection (hpi). Cells infected with EV71 and CA16 at 0 hpi were used as controls.

African green monkey kidney (Vero) cells used for viral plaque assays were obtained from IMBCAMS (Kunming, China) and grown in minimum essential medium with 10% newborn bovine serum (Gibco, USA), L-glutamine, and 1% penicillin/streptomycin at 37 °C in a 95% air and 5% CO_2_ atmosphere.

293T cells for the dual-luciferase reporter assay were provided by IMBCAMS (Kunming, China) and were cultured in Dulbecco’s modified Eagle medium containing 10% fetal bovine serum and 1% penicillin/streptomycin solution in a humidified incubator at 37 °C with 5% CO_2_.

### Virus titration of EV71 and CA16 in HUVECs

A plaque assay was used to quantify infectious viruses recovered from HUVECs at the abovementioned timepoints. Briefly, the harvested cells were disrupted by three freeze-thaw cycles, and serial dilutions of all samples were inoculated into confluent Vero cells on 6-well plates. After 3 h of incubation to allow virus attachment, the wells were gently washed with PBS, covered with 2 mL of minimum essential medium containing 1% agarose, and inverted into a 37 °C CO_2_ incubator for 48 h. Next, the cells were fixed with 2 mL of 4% paraformaldehyde (Solarbio, China) at 15 °C to 25 °C for 30 min, and the 1% agarose was removed. The monolayer of cells was stained with a crystal violet staining solution for 15 min and washed with ddH_2_O. Finally, visible plaques were counted by the naked eye, and the plaque-forming units (pfu/mL) were calculated with the virus titer formula, in which the virus titer equals the number of plaques × (1 mL) × dilution factor.

### Measurement of HUVEC permeability

HUVECs were grown to confluent monolayers on clear polyester Transwell filters (0.4 mm pore size, 12 mm diameter; Corning, USA). After completely treating with EV71 and CA16, we assessed the integrity and uniformity of the HUVEC monolayers with an In Vitro Vascular Permeability Assay Kit (Millipore, USA). Briefly, the medium was carefully removed from the Transwell insert, and 150 μL of FITC-dextran was added to the top chamber for incubation at room temperature for 20 min in the dark. At the end of the incubation period, the FITC-dextran content of the lower chamber was measured using a fluorescence plate reader (Bio-Rad, USA) at an excitation wavelength of 485 nm and an emission wavelength of 530 nm.

### Flow cytometry assay

Cell apoptosis evaluation was performed by flow cytometry. The harvested cells were washed with PBS and resuspended at a density of 1 × 10^6^ cells/mL in 1 × Annexin V binding buffer followed by thorough mixing. After double staining with 5 μL of Annexin V-FITC and 5 μL of propidium iodide per sample for 15 min at 25 °C in the dark using the Annexin V-FITC Apoptosis Detection Kit (Beyotime, China), the cells were analyzed using a FACScan^®^ flow cytometer equipped with Cell Quest software (BD Biosciences, USA), according to manufacturer’s instructions, to detect early and late apoptosis of cells.

### Bioinformatics analysis of miRNA sequencing data

According to previous bioinformatics analysis methods, principal component analysis, differential expression analysis of miRNAs, unsupervised hierarchical clustering, trend analysis, target prediction, gene ontology (GO) and pathway enrichment of differentially expressed miRNAs, and coexpression network analysis of key target genes were systematically conducted study utilizing the corresponding software^33^. The sequencing data were deposited in the National Center for Biotechnology Information’s Gene Expression Omnibus (GEO) database (www.ncbi.nlm.nih.gov/geo/) and are accessible at series number GSE94551.

### Quantitative reverse transcription-polymerase chain reaction (qRT-PCR) for viral load and miR-1303 and MMP9 expression

For viral load examination, total RNA was extracted from all samples using the standard TRIzol reagent (TIANGEN, China) protocol. The regions of standards generated from EV71-VP1 and CA16-VP1 RNA were amplified by the PrimeSTAR^®^ HS reagent (TAKARA, Japan) with the primers shown in Table S1. The target 624-bp region (EV71-VP1) and 682-bp region (CA16-VP1) were cloned into the pGM-T vector (TIANGEN, China) following the manufacturer’s guidelines. A single recombinant clone containing the insert under the T7 promoter was selected, and the insert was transcribed in vitro using the TranscriptAid T7 High Yield Transcription Kit (Thermo Scientific, USA) according to the manufacturer’s recommendations. The in vitro-transcribed RNA was purified using the GeneJET RNA Purification kit (Thermo Scientific, USA) and quantified spectrophotometrically using the Nanodrop 2000 (Thermo Scientific, USA). The qRT-PCR process was performed using a One Step PrimeScript™ RT-PCR Kit (TAKARA, Japan). To generate a standard curve for cycle thresholds (Cts) versus virus copy number, the purified RNA was serially diluted to known concentrations in the range of 10^1^ to 10^8^ copies/μL and assayed concurrently with the test samples. The PCR amplification cycle was set at 42 °C for 5 min for reverse transcription, followed by 95 °C for 10 s for initial denaturation and 40 cycles of 5-s denaturation at 95 °C and 34-s annealing at 60 °C. The reactions were run on a 7500 Fast Real-time PCR System (Applied Biosystems, USA).

For the miR-1303 analysis, miRNA was enriched by a miRcute miRNA Isolation Kit (TIANGEN, China). Subsequently, 2 μg of RNA was first polyadenylated with polyA polymerase and reverse-transcribed into cDNA with a poly(T) adapter primer under the following reaction conditions: 60 min at 37 °C, 5 min at 85 °C, and maintenance at 4 °C. Finally, PCR was performed using SYBR Premix Ex Taq (TAKARA, Japan) in accordance with the manufacturer’s protocols, on an ABI7500 real-time PCR system (Applied Biosystems, USA). The amplification conditions were as follows: 95 °C for 10 s, 40 cycles of 95 °C for 10 s, and 60 °C for 40 s, and dissociation at 95 °C for 60 s, 55 °C for 30 s, and 95 °C for 30 s. The qRT-PCR results were calculated with the comparative threshold cycle (Ct) method and U6 snRNA, which was provided with the kit and served as an internal control. The primers used in this study are listed in Table S1. The relative amount of expressed miRNAs and mRNAs was also calculated by the 2^−ΔΔCt^ method.

### Dual-luciferase reporter assay

The miR-1303 normal and mutated seed sequences in the 3′-untranslated (UTR) of the MMP9 gene were chemically synthesized in vitro (Sangon Biotech, China), and the Xho I and Not I restriction sites were added to both ends. The two DNA fragments were cloned into the psiCHECK-2 luciferase reporter plasmid (Promega, USA) to generate a wild-type (WT) MMP9-3′UTR plasmid and a mutant MMP9-3′UTR plasmid. For the dual-luciferase reporter assay, 293T cells were seeded into 96-well plates and transfected with different combinations of miR-1303 mimics, miR-1303 inhibitor, negative control (NC) plasmid, NC inhibitor, WT MMP9-3′UTR plasmid, and mutant MMP9-3′UTR plasmid using Lipofectamine 2000 (Promega, USA), strictly according to the manufacturer’s instructions. After 48 h of incubation, the cells from each group were lysed, and the fluorescence intensity was measured with a GloMax 20/20 luminometer (Promega, USA) using Renilla luciferase as the internal reference.

### Enzyme-linked immunosorbent assay (ELISA)

Cell samples were centrifuged at 3000 × *g* for 10 min at 4 °C, and supernatants were assessed using a commercial MMP9 ELISA kit (Abcam, USA) according to the manufacturer’s recommendations. In microplates, standards (50 µL), samples (10 µL sample liquid and 40 µL diluent), and blanks were added into predefined wells and incubated for 1 h at room temperature. After the plates were washed twice with wash buffer, 100 µL of horseradish peroxidase (HPR)-labeled conjugates was added prior to sealing the plates for incubation at 37 °C for 1 h. Following five stringent washes, substrates A (50 µL) and B (50 µL) were added into each well for incubation at 37 °C for 15 min. Eventually, a stop solution (50 µL) was added into each well, and the absorbance of each well was measured at 450 nm within 15 min using a microplate reader (Thermo Fisher, USA). MMP9 levels in these samples were located within the linear range of the standard curves.

### Western blot analysis

Cultured cells and tissue specimens were homogenized with radioimmunoprecipitation assay lysis buffer containing 50 mM Tris-HCl (pH 7.5), 150 mM NaCl, 0.1% Nonidet P-40, and a mixture of protease inhibitors for 20 min on ice. Further, the lysates were centrifuged to remove the cellular debris, and protein concentrations were measured with the Bradford protein assay (Thermo Fisher, USA) following the manufacturer’s protocols. Equal amounts of protein (30 μg) were subjected to 8–10% sodium dodecyl sulfate polyacrylamide gel electrophoresis based on their molecular weight and transferred to a PVDF membrane (Millipore, USA). After blocking with 5% skim milk in Tris-buffered saline with 0.1% Tween-20 solution for 2 h at room temperature, the membrane was incubated with primary antibodies against MMP9 (1:1000 dilution), Claudin4 (1:1500 dilution), Claudin5 (1:1500 dilution), VE-Cadherin (1:1200 dilution), ZO-1 (1:2000 dilution), and GAPDH (1:500 dilution; as a loading control) at 4 °C overnight. Antibodies specific to the above proteins were obtained from Abcam, USA. Subsequently, the blot was thoroughly washed with TBST three times for 15 min each and incubated with HRP-conjugated secondary antibodies (1:12,000 dilution; Abmart, USA) for 1 h at room temperature. Immunoreactive bands were developed with an enhanced chemiluminescence kit (Beyotime, China) following the manufacturer’s manual and then detected with a ChemiDoc™ Imaging System (Bio-Rad, USA). Finally, the gray values of the protein bands were quantified using ImageJ software.

### Immunofluorescence (IF) microscopy

HUVECs were seeded onto poly-L-lysine-coated coverslips (Solarbio, China). At the indicated time, the cells were washed twice with PBS, fixed with 4% paraformaldehyde for 30 min, and permeabilized with 1% Triton X-100 in PBS for 15 min at room temperature. After blocking with 5% bovine serum albumin, the cells were incubated with the primary antibodies in blocking solution against EV71/CA16-VP1 (1:1000 dilution; Millipore, USA), Nectin1 (1:100 dilution), Claudin4 (1:200 dilution), VE-cadherin (1:500 dilution), and ZO-1 (1:200 dilution) overnight at 4 °C. Next, the cells were washed with PBS three times and then incubated with Alexa Fluor 594-conjugated donkey anti-rabbit IgG (1:1000 dilution; Abcam, USA) or Alexa Fluor 647-conjugated donkey anti-mouse IgG (1:1000 dilution; Millipore, USA) for 1 h at room temperature. The nuclei were counterstained with 4′, 6-diamidino-2-phenylindole (DAPI, 1:4000 dilution; Beyotime, China). Following three washes in PBS, the coverslips were mounted on slides with an antifade reagent (Solarbio, China). Finally, images of the cells were captured with a laser-scanning confocal microscope (Leica, Germany) and processed using Adobe Photoshop 7.0 software.

### Hematoxylin and eosin staining

After sacrifice, a rhesus monkey was necropsied to obtain lung and brain tissues. All the tissue samples were fixed in 10% formalin at pH 7.4, embedded in paraffin, and sectioned with a microtome into 5-μm-thick sections. The sections were deparaffinized in xylene, dehydrated with graded ethanol, and washed three times with PBS (5 min per wash). Subsequently, the sections were subjected to hematoxylin and eosin staining to evaluate the damage to the lung and brain tissues. Finally, the slides were passed through stepwise dehydration in increasing concentrations of ethanol (70–100%), 5 min for each step, followed by 5~10 min in xylene. The sections were observed and photographed under a light microscope (Leica, Germany).

### Statistical analysis

The data are expressed as the means ± standard deviation of experiments repeated at least three independent times and were analyzed with SPSS 20.0 software (IBM, USA). Two-tailed Student’s *t*-tests were used for comparisons between two groups. Multigroup measurement data were analyzed using one-way analysis of variance. All *P* values were two-sided, and a *P* value less than 0.05 was considered statistically significant.

## Electronic supplementary material

Table S1

Figure S1

Figure S2

Figure S3

Figure S4

Figure S5

Figure S6

Figure S7

Figure S8
