# Supplementary material for: miR-1303 regulates BBB permeability and promotes CNS lesions following CA16 infections by directly targeting MMP9
Source: Emerg Microbes Infect. 2018 Sep 19;7:155. doi: 10.1038/s41426-018-0157-3 (PMC6143596; doi:10.1038/s41426-018-0157-3)
Supplement: Supplementary file 1 — Table S1 [file 41426_2018_157_MOESM1_ESM.docx]

**Table. S1 primers and probes used in the study.**

| **Genes** | **Primers and probes sequences** |
| --- | --- |
| EV71-VP1-1(Standard plasmid construction) | 5’-TGCCAACTGGGACATAGATATAACAGG-3’ (sense)  5’-ACTCTAAAGTTGCCCACATAAATAGCC-3’(anti-sense) |
| EV71-VP1-2 (qRT-PCR detection) | 5’-ACCTATTCAAAGCCAACCCAA-3’ (sense)  5’-TAAATAGCCCCGGACTGTTGT-3’(anti-sense)  Probe: FAM-TTTCCCAAGAGTGGTGATCGCTGT-TAMRA |
| CA16-VP1-1(Standard plasmid construction) | 5’-AACACTGAGGCTAGTAGTCAC-3’ (sense)  5’-CGTGTTTGATTCTCATGTACACC-3’(anti-sense) |
| CA16-VP1-2 (qRT-PCR detection) | 5’-GTTTGTGAAAATGACGGACCC-3’ (sense)  5’-GTCATTTGCTTGAAGGTGCTC-3’(anti-sense)  Probe: FAM-CAGCTCAAGTGTCAGTCCCCT-TAMRA |
| miR-1303 | 5′-GTTTAGAGACGGGGTCTTG-3′(sense) |
| MMP9 | 5′-CAGTACCACGGCCAACTACGACACC-3′(sense)  5′-CAGTACCACGGCCAACTACGACACC-3′(anti-sense) |
