# Supplementary figures and images for: miR-1303 regulates BBB permeability and promotes CNS lesions following CA16 infections by directly targeting MMP9
Source: Emerg Microbes Infect. 2018 Sep 19;7:155. doi: 10.1038/s41426-018-0157-3 (PMC6143596; doi:10.1038/s41426-018-0157-3)

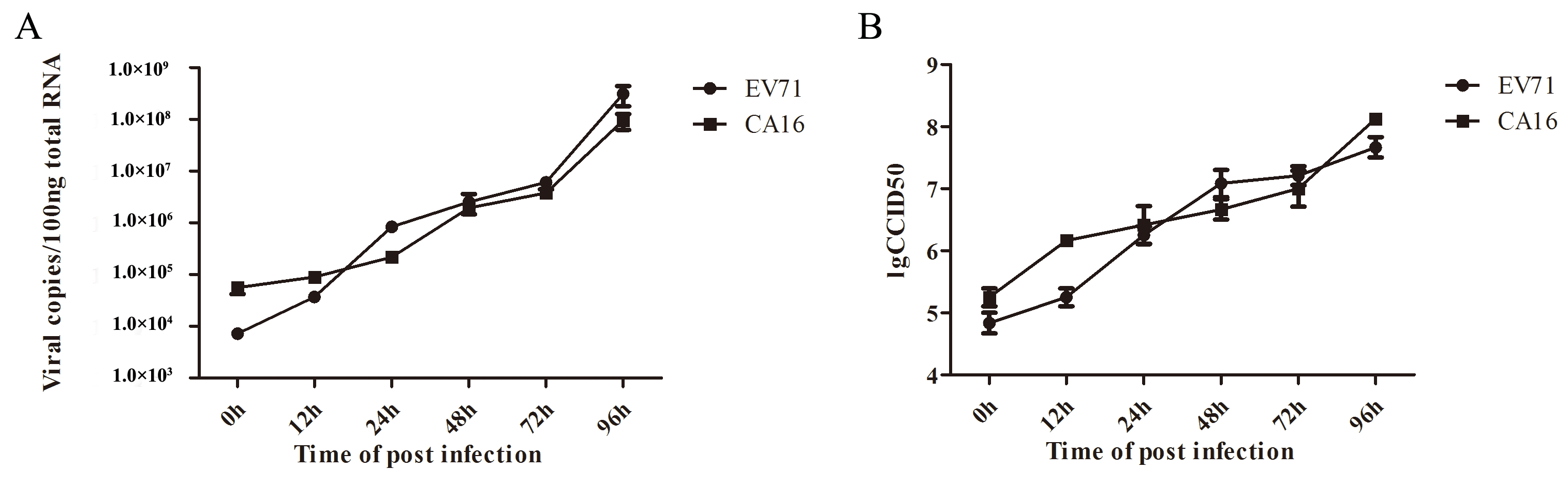

Supplement: Supplementary file 2 — Figure S1 [file 41426_2018_157_MOESM2_ESM.tif]

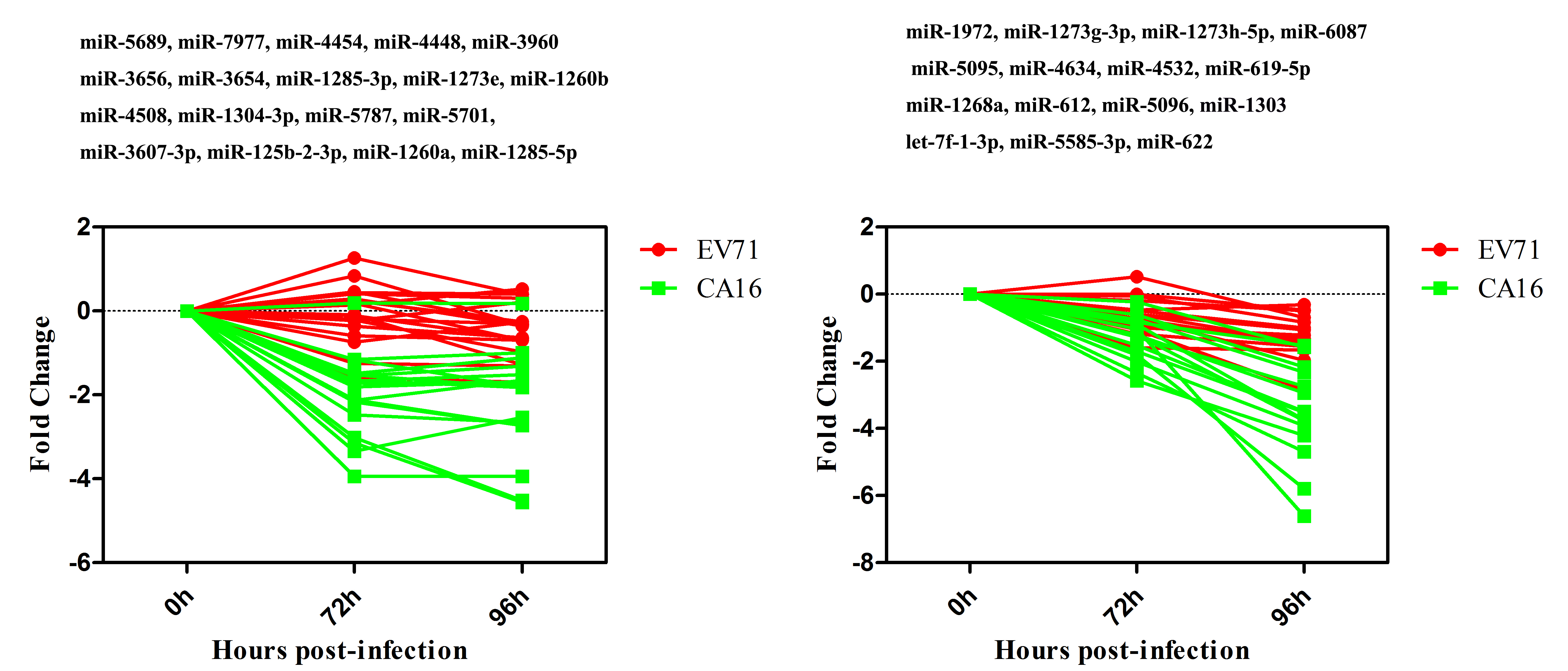

Supplement: Supplementary file 3 — Figure S2 [file 41426_2018_157_MOESM3_ESM.tif]

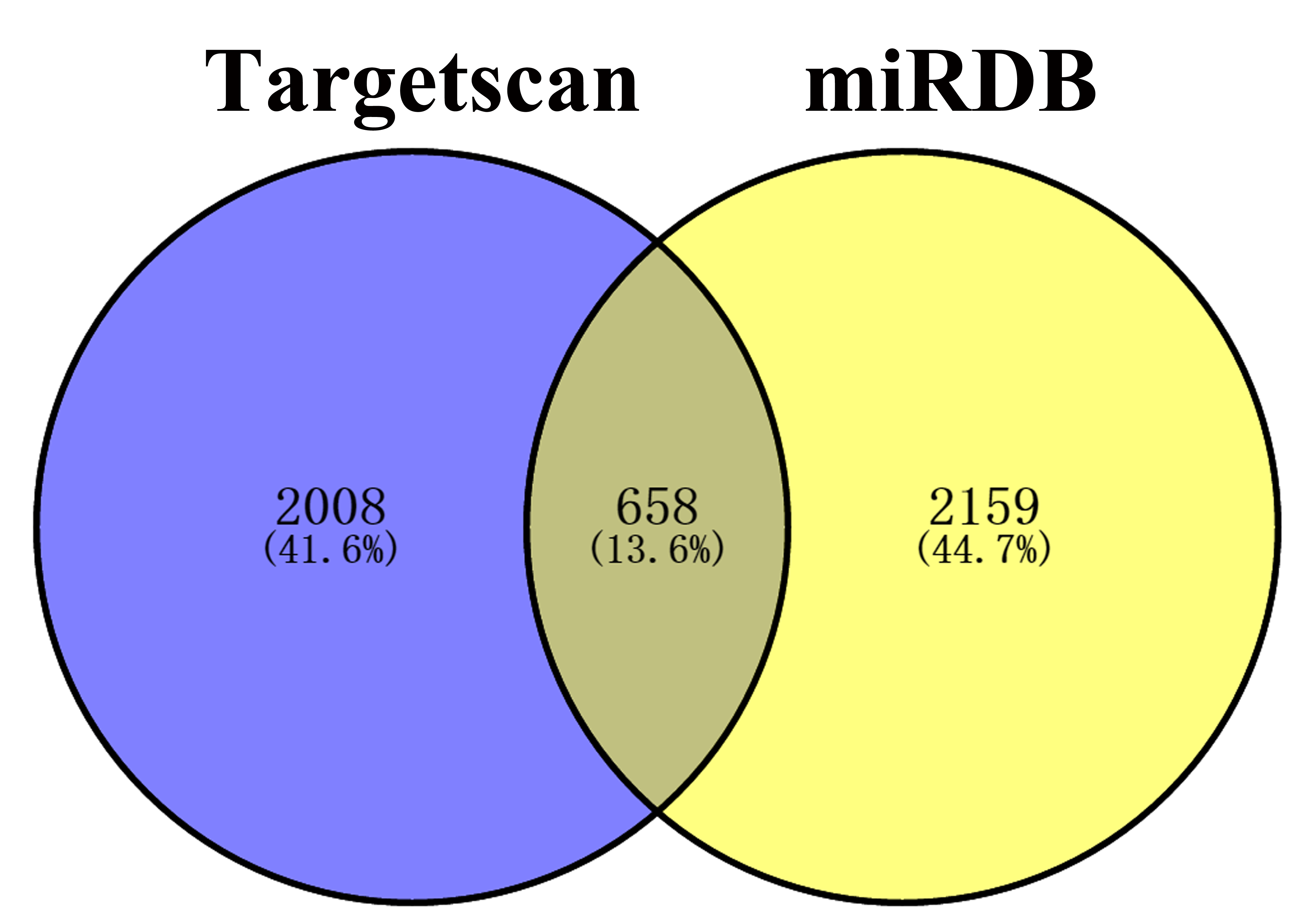

Supplement: Supplementary file 4 — Figure S3 [file 41426_2018_157_MOESM4_ESM.tif]

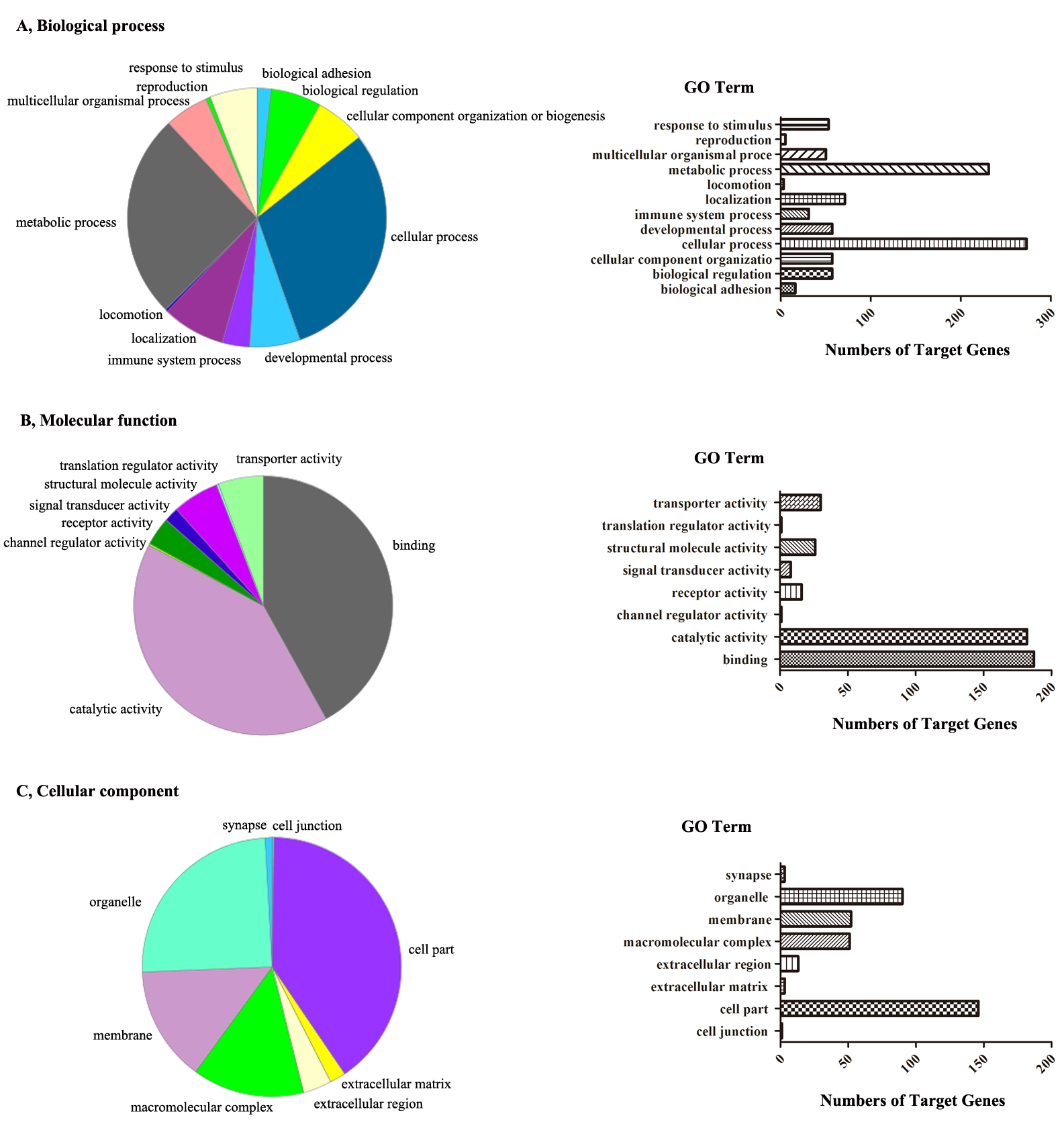

Supplement: Supplementary file 5 — Figure S4 [file 41426_2018_157_MOESM5_ESM.tif]

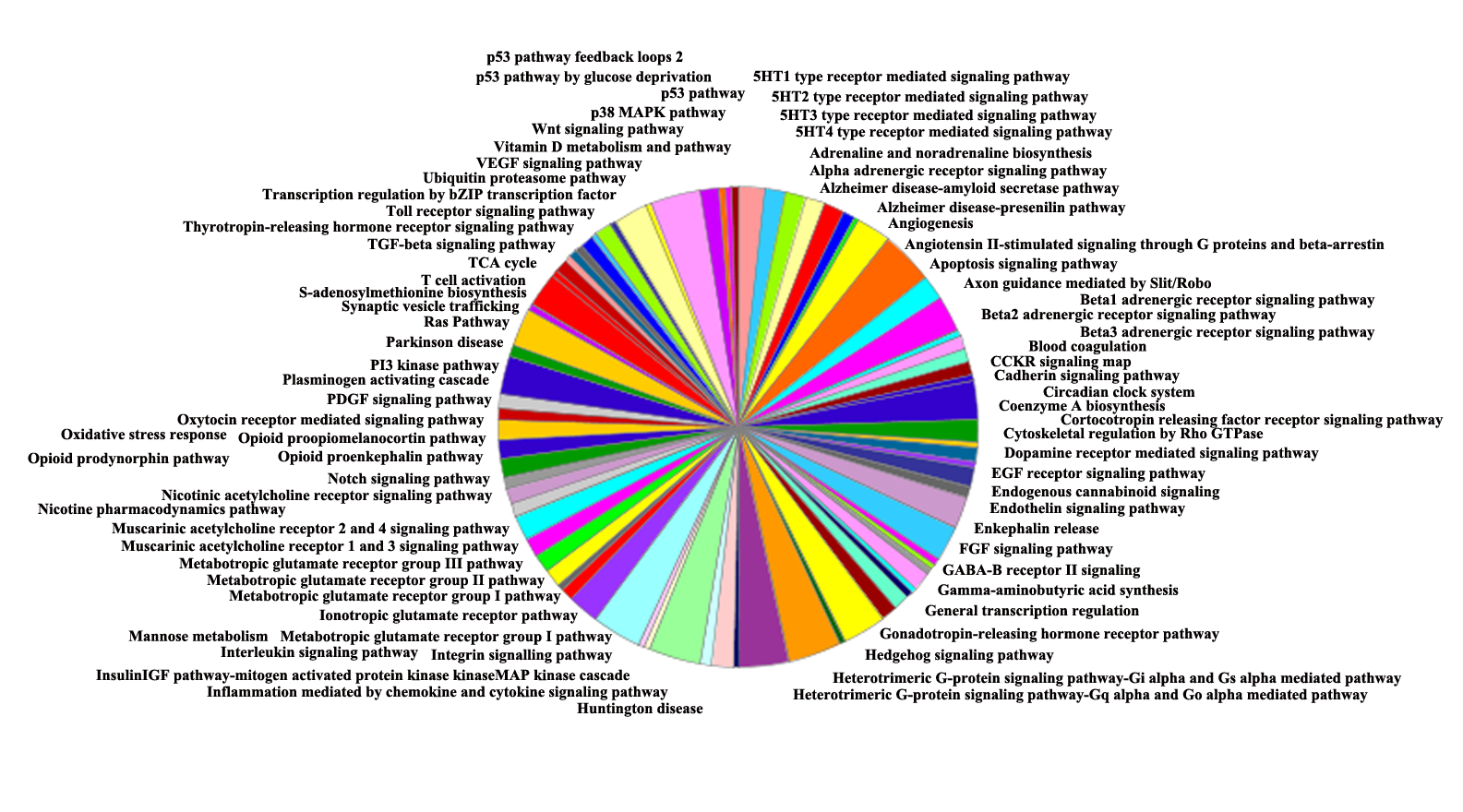

Supplement: Supplementary file 6 — Figure S5 [file 41426_2018_157_MOESM6_ESM.tif]

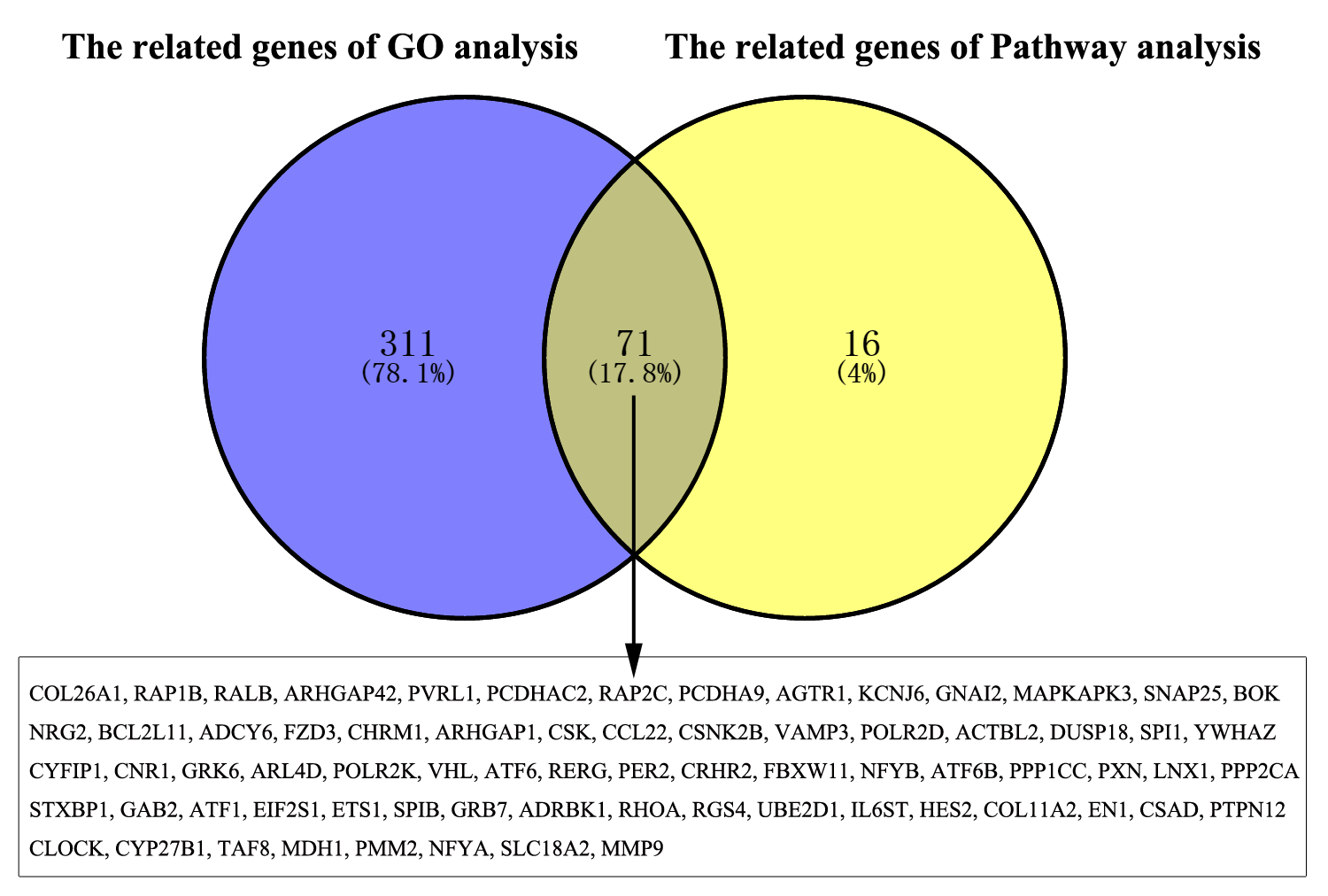

Supplement: Supplementary file 7 — Figure S6 [file 41426_2018_157_MOESM7_ESM.tif]

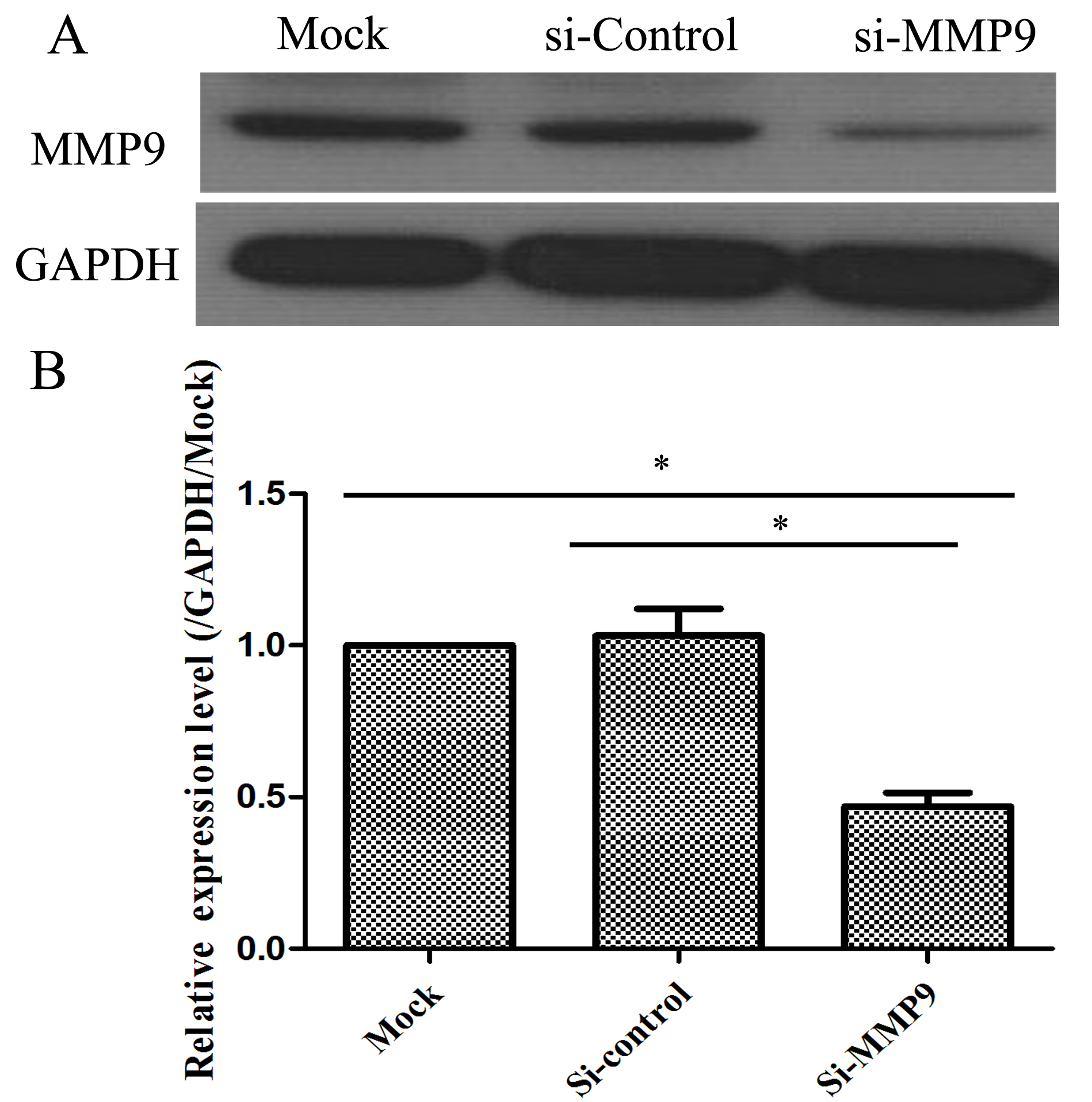

Supplement: Supplementary file 8 — Figure S7 [file 41426_2018_157_MOESM8_ESM.tif]

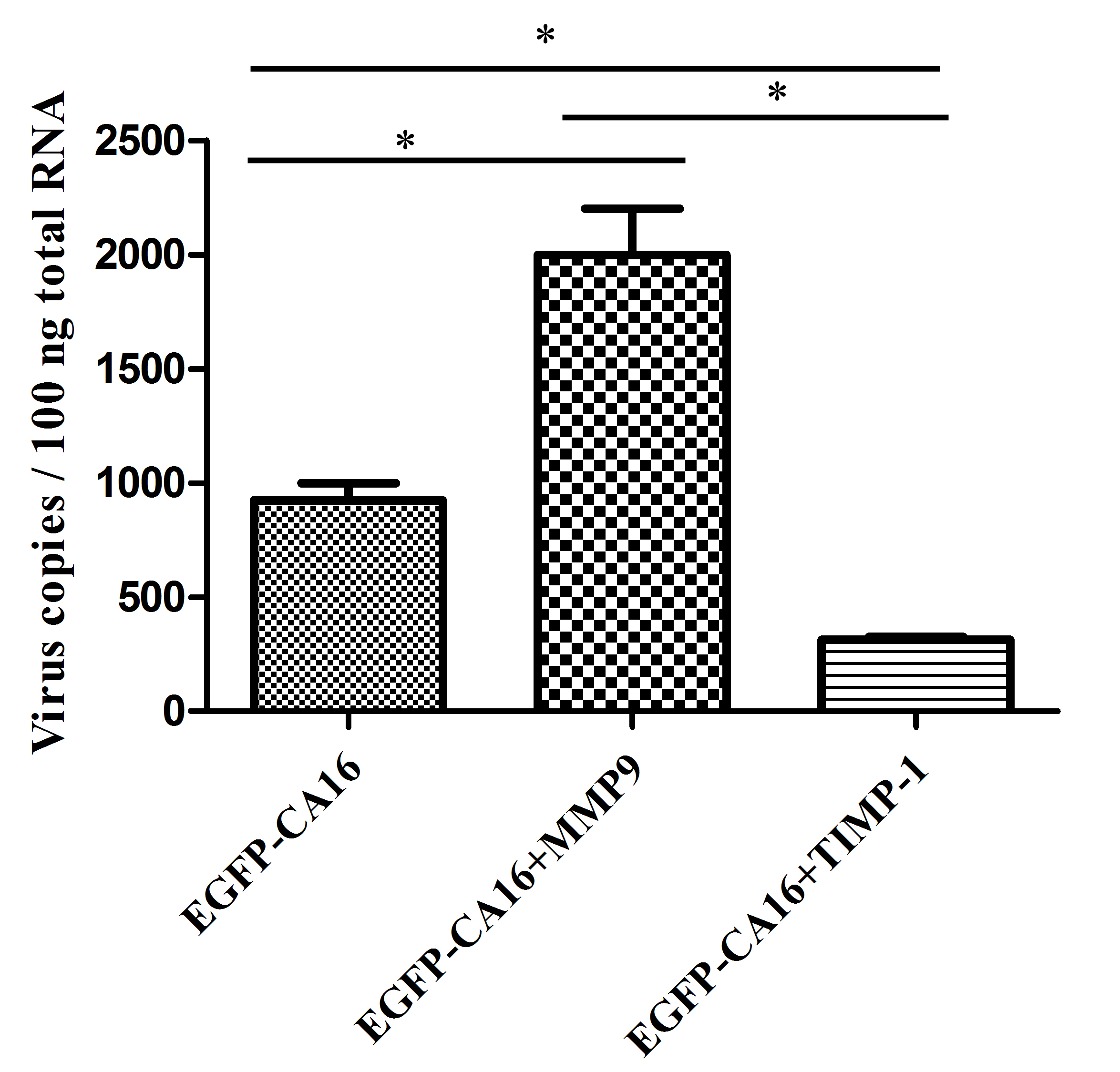

Supplement: Supplementary file 9 — Figure S8 [file 41426_2018_157_MOESM9_ESM.tif]
